# Preliminary evaluation of a scenario-based nutrition literacy online programme for college students: a pilot study

**DOI:** 10.1017/S1368980023002471

**Published:** 2023-12

**Authors:** I-Ju Lai, Li-Chun Chang, Chia-Kuei Lee, Li-Ling Liao

**Affiliations:** 1 Department of Nutrition, I-Shou University, Kaohsiung, Taiwan; 2 Department of Nursing, Chang Gung University of Science and Technology, Gueishan, Taiwan; 3 Department of Nursing, College of Medicine, National Cheng Kung University, Tainan, Taiwan; 4 Department of Public Health, College of Health Science, Kaohsiung Medical University, No.100, Shih-Chuan 1st Road, Sanmin Dist., Kaohsiung City, Taiwan, 80708, Taiwan

**Keywords:** Nutrition literacy, College students, Scenario-based approach, Self-directed online learning

## Abstract

**Objective:**

This study aimed to develop and evaluate a scenario-based nutrition literacy (NL) online programme for Taiwanese college students.

**Design:**

A randomised pilot trial design was used in this study.

**Setting:**

The study was conducted at a university in Taiwan. The intervention consisted of a five-unit web-based NL programme including videos of real-life scenario-based stories, situational analysis teaching and after-unit quizzes. Theme-related website information and smartphone apps (both iOS and Android systems) were offered for reference in every unit. The NL measure consisted of a self-rated scale, a scenario-based test and a healthy eating behaviour survey. Paired sample t-tests and ANCOVA were performed to test the effects on NL and healthy eating behaviour.

**Participants:**

Participants were ninety-eight students, with a retention rate of 98 %. The ratio of men to women was 0·2:1. Most students were freshmen (48 %).

**Results:**

Compared with the control group, the experimental group showed significant post-intervention improvements in the NL and healthy eating behaviours after controlling for pretest scores.

**Conclusions:**

This pilot study offers preliminary evidence of the potential positive effects of implementing a scenario-based NL online programme for college students. It offers a possibly novel strategy to enhance health-promoting behaviours in Taiwanese universities. Further research with larger sample sizes and more rigorous designs is warranted to confirm and build upon these initial findings.

During the transition to college life, young adults undergo significant changes in their diet and eating patterns, making it crucial to establish healthy eating habits^(1)^. Research conducted in various regions, including Taiwan, consistently reveals that college students’ eating behaviours are often suboptimal^(2–4)^, placing them at risk of rapid weight gain and compromised nutritional status with potential long-term health consequences^(5)^. To address these challenges, nutrition education emerges as a vital solution, employing various strategies to encourage healthy eating choices and positive nutrition-related behaviours^(6)^. Notably, nutrition education has proven effective in improving college students’ dietary habits^(7,8)^. Therefore, targeted interventions for this group are essential to enhance their overall health and well-being.

Nutrition literacy (NL) is a type of health literacy that emphasises obtaining, processing and understanding the nutrition information and skills needed to make appropriate nutrition-related decisions^(9,10)^. Limited health literacy and NL are associated with nonadherence to healthy diet patterns^(11)^, low diet quality, high sugar-sweetened beverage intake^(12)^ and a lack of knowledge about food label use and interpretation^(13)^ and food portion calculation^(14)^. Moreover, higher levels of NL are positively associated with healthier eating behaviours^(4)^. NL appears to play a significant role in influencing individuals’ dietary habits. Existing NL intervention studies primarily focus on children^(15)^ and adolescents^(16)^ or parents^(10,17)^. A 2022 scoping review^(16)^ revealed considerable heterogeneity in current NL interventions for children and adolescents, including intervention types, evaluation methods and participants. Furthermore, it also identified certain favourable factors contributing to the effectiveness of NL interventions, such as incorporating technological components, using multiple modalities, and extending intervention duration beyond 4 weeks. Despite extensive research on nutrition interventions in public health for college students^(18)^, there is a scarcity of studies specifically targeting college students’ NL. NL is a relatively new concept still under discussion^(19)^, and many health educators and professionals might not have a complete understanding of NL and how it affects nutrition education, which could make it challenging to incorporate it into existing programmes. To fully leverage the importance of NL, additional strategies and examples for integrating it into nutrition education are needed.

Combining scenario-based learning with an NL-based intervention is crucial for successful nutrition education, as it enables meaningful practice in real-life situations. Grounded in situational learning theory^(20)^, this approach emphasises the significance of education within authentic contexts, fostering meaningful learning experiences and enhancing subject understanding. It represents a contemporary and effective way to educate students, allowing them to grasp and retain knowledge practically^(21)^. Real-life scenario-based instruction is widely used in various professional fields, such as teaching^(22)^ or medicine^(23)^, and addressing educational issues for adolescents^(21,24)^. Empirical studies mainly focus on educational training interventions, consistently showing positive outcomes. This method effectively prepares learners for practical application while boosting self-efficacy and professional readiness in their respective domains^(22)^. By adopting a socio-cognitive perspective^(25)^, self-efficacy refers to the belief that individuals possess the ability to cope effectively with challenging situations. By incorporating the principle of self-efficacy into scenario-based instruction, educators can create a conducive learning environment that empowers learners to face future challenges with confidence and determination.

The rise of digital technologies has led to the emergence of online platforms for implementing nutrition education interventions. These platforms utilise the Internet to enhance accessibility, cost-effectiveness and flexibility^(26)^. A systematic review investigating effective online nutrition education interventions^(27)^ has highlighted their potential and attracted considerable attention. These interventions aim to assess the feasibility and impact of using online platforms across diverse age groups, including children, adolescents and adults. Among the 27 studies analysed in this review, nine studies specifically targeted college students or young adults. The findings from these studies suggest that online interventions can effectively disseminate nutritional information and promote positive changes in dietary choices in this demographic group.

Thus, we developed a scenario-based online intervention to improve NL and healthy eating behaviours among Taiwanese college students, whose NL was likely to be suboptimal^(4)^. This pilot study was conducted to evaluate the effectiveness of the novel intervention.

## Materials and methods

### Study design

This study was a randomised pilot trial. The experimental group received a 5-week NL online intervention programme between April and May 2018, including a 30-min lesson/week, whereas the control group did not receive any nutrition education intervention. Each participant in the experimental group was assigned a set of accounts and passwords to log into the course and complete it within the specified time. The research assistant would send an email or text message to remind the participants when each unit of the online course was open and whenever the participants had not completed the online course within the time limit. All students who took part in the research project completed all online courses. Both groups were asked to complete the pretest and post-test online questionnaires prior to and after completion of the online programme, respectively.

### Participants and recruitment

To determine the sample size for this pilot study, recommendations from the relevant literature were adopted. Various studies have recommended overall trial sample sizes of 24–70 participants^(28–30)^. Moreover, one study recommended over thirty participants/group for pilot studies^(31)^. Considering these prior recommendations, our aim was to have a minimum of 30 participants per group. To account for the potential dropout rate, which was estimated to range between 40 % and 80 % among online learners^(32)^, we calculated a sample size of fifty participants per group by dividing 30 by 60 % (0·6) due to this estimated dropout rate estimation, while also considering budget constraints.

To be eligible for this study, participants had to be students of a private university in Southern Taiwan. The recruitment process was facilitated by the school’s health centre through emails sent to all students and LINE messages to members of the health centre’s volunteer group, which comprised volunteers from various departments. These members helped disseminate information through personal messages or face-to-face interactions, and their involvement was not limited to their respective departments. Recruitment posters were posted on campus bulletin boards in the meantime. Consequently, a total of 100 students who volunteered to participate were recruited and randomly allocated to the experimental and control groups, with fifty students per group. The random allocation was conducted using a computer-generated process, employing a random number generator to assign participants to either the experimental or control group. This method ensured unbiased assignment and minimised potential systematic biases in participant allocation. As the recruitment was based on the students’ free will, the participants were from thirty-one departments of the university, such as the Department of Accounting, Department of Tourism, Department of Electronics and Department of Management. After group randomisation, the research team sent individual participants an email explaining the research schedule, process, friendly reminders and remuneration. The incentives for the experimental and control groups were NT$ 500 (approximately US$ 16·8) and NT$ 100 (approximately US$ 3·4), respectively. Overall, two students withdrew mid-study owing to personal issues, and ninety-eight students completed the study (98 % retention rate).

### Intervention

**Fig. 1:**
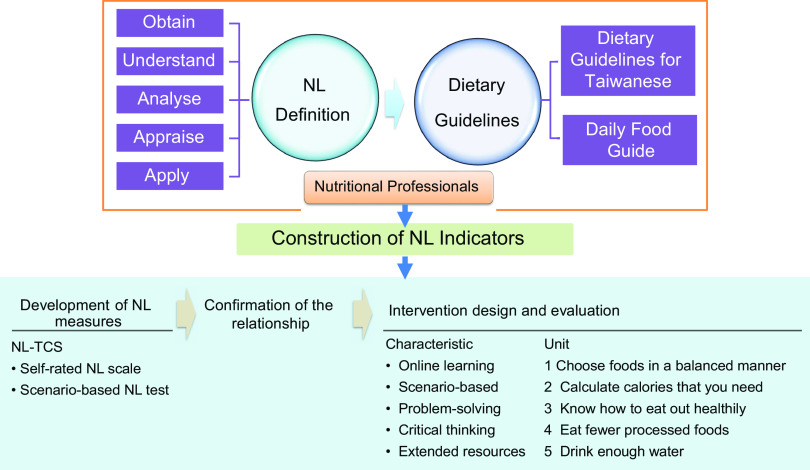
Framework of constructing nutrition literacy (NL) indicators for Taiwanese college students

This study was part of the research project that aimed at promoting NL among Taiwanese college students and consisted of four parts: (1) constructing NL indicators^(33)^; (2) developing NL measures^(34)^; (3) confirming the relationship between NL and healthy eating behaviours^(4,35)^; and (4) conducting a pilot interventional research to enhance college students’ NL. Figure 1 shows the framework of the research project. The results of the first three parts have been published elsewhere^(4,33–35)^, and this study presents the results of the fourth part. The development of the intervention programme partially used the results of the first and second parts, which are briefly described below.

### Development of principal indicators

NL indicators for Taiwanese college students were used as the main principle to develop the content of the NL online programme. The indicators were built with a two-round Delphi survey to gradually agglomerate a consensus from dietitians and nutritionists on NL indicators for Taiwanese college students. The consensus was reached on eight principal indicators based on the five domains of the holistic connotation of NL, with two *obtain* (able to search for, find, and acquire nutrition information), two *understand* (having basic nutrition knowledge and the capacity to understand general nutrition information), one *analyse* (able to discriminate and analyse nutrition information in a given situation), two *appraise* (able to judge and assess nutrition information in terms of personal needs) and one *apply* item (able to apply nutrition information to daily life to achieve a healthy diet). A total of twenty-eight sub-indicators were designed based on the Dietary Guidelines for Taiwanese within eight themes, including ten in *understanding*, eight in *analysis*, five in *appraisal* and five in the *application* domain. Detailed information regarding the NL indicators used in this study has been published elsewhere^(33)^.

### Intervention programme

The NL online intervention programme consisted of five units, each taking approximately 30 min to complete. The learning objectives for each unit were derived implicitly from the corresponding NL domains and indicators (details in Table 1). Upon programme completion, students were expected to reach the ideal NL level for Taiwanese college students, covering 28 sub-indicators across eight themes. The learning content focused on common dietary issues faced by Taiwanese college students. Scenario-based instruction aimed to replicate real-life behaviours and foster immediate application of learned content, promoting successful experiences^(36)^. Each unit included a 5-min video depicting a real-life scenario performed by students on campus, followed by a 15-min situational analysis focusing on the protagonist’s dietary choices. Subsequently, a post-unit quiz with a maximum of 10 questions (sample shown in Table 2) was administered. The quiz was accessible only after completing the first two components and served as a self-assessment tool for learners to gauge their understanding and comprehension of the unit’s learning objectives. Participants received feedback on incorrect answers and needed to achieve a perfect score to successfully complete the unit and ensure intended learning outcomes. To fulfil the learning task, participants were required to complete all three parts, and the intervention plan mandated completing the previous part to proceed. The flexible completion time accommodated each learner’s pace. Additionally, each unit integrated theme-related information from official and trustworthy non-governmental organisation websites and smartphone apps (iOS and Android systems) to provide students with accurate nutrition-related information after class.

**Table 1:** Learning objectives and contents of the NL online intervention programme

Unit	Learning objectives corresponding to NL domains and indicators	Real-life scenario/learning content
1. Choose foods in a balanced manner	【Understand】 1-1-1 Understand the meaning of the Daily Food Guide 1-1-2 Understand the classification of the six food groups and dietary values 1-1-3 Understand the influence of well-balanced diets on health 1-5-1 Understand the influence of adequate intake of whole-grain foods on health 【Analyse】 2-1-1 Analyse the types of foods from six groups and the recommended quantity of an individual’s daily diet 2-5-1 Analyse the types and quantity of whole-grain foods an individual should consume daily 【Appraise】 3-1-1 Appraise whether an individual’s daily diet conforms to the recommended types and quantity of foods from the six groups 【Apply】 4-1-1 Choose or modify personal diet based on the recommended types and quantity of foods from six groups	【Scenario】 Use the myths and misinformation related to college students’ daily meals as the background. Example: Ray did not eat anything more than the custard bread he bought in the convenience store in the morning. In the evening, Ray just bought a bottle of flavoured milk to have while attending club activities. Afterwards, Ray felt very hungry, so he decided to go to the night market for supper. 【Learning content】 Connect with the daily diet of college students and teach them how to use the daily diet guide to choose three meals considering deliciousness and nutritional balance as factors.
2. Calculate calories that you need	【Understand】 1-2-1 Understand the relationship between healthy body weight and risk for chronic diseases 1-2-2 Understand the definitions of healthy body weight and calculation of BMI 1-3-1 Understand the purpose of physical activity 【Analyse】 2-2-1 Analyse personal healthy body weight and energy requirements 【Appraise】 3-2-1 Appraise whether an individual’s daily diet conforms to personal energy requirements 3-3-1 Appraise whether an individual’s physical activity is appropriate and adequate 【Apply】 4-2-1 Choose or modify personal diet based on personal energy requirements 4-6-1 Choose the low-oil cooking method and dishes for personal health reasons	【Scenario】 Set the weight loss plan of college students as the background; the story will contain various Internet rumours about weight loss. Example: In order to lose weight, Ray used the fasting method introduced on the Internet. Because he was hungry and almost fainted, he used weight loss pills and drank weight loss drinks instead. However, he experienced palpitations, chest tightness, dizziness, and cold sweats. 【Learning content】 Motivate students by ‘busting’ internet rumours. Explain the meaning of ‘calories’ and teach them how to design personal weight control programmes using simple steps.
3. Know how to eat out healthily	【Understand】 1-11-1 Understand food hygiene and safety precautions when buying foods (such as reading food labels and sources of production) 【Analyse】 2-2-2 Analyse the energy of packaged foods based on nutrition facts 2-6-1 Analyse sodium content of packaged foods based on nutrition facts 2-6-2 Analyse types of high-sodium foods eaten in daily life 2-6-3 Analyse types of high calories, low-nutrient density foods	【Scenario】 After college students start to live outside of the family home, their chances of eating out every day also increase. Use the notion that college students choose unhealthy foods when faced with a variety of food choices as the story. Example: Ray likes to buy street-food snacks and often goes to convenience stores to buy snacks or microwave foods for three meals. When meeting with friends, he prefers buffets, but he never cares about food hygiene or eating a high-sodium diet. 【Learning content】 Use various food selection environments, such as convenience stores, night markets and cafeterias, as the story background to teach the principles and steps related to choosing healthy and nutritious foods in various situations.
4. Eat fewer processed foods	【Appraise】 3-8-1 Appraise whether an individual’s diet contains too many excessively processed foods 【Apply】 4-8-1 Choose foods that are not excessively processed for personal health reasons 4-8-2 Choose plant foods more often for personal health reasons	【Scenario】 The background of the story is the choice between various natural ingredients and processed foods that college students face when eating at hotpots. Example: Ray found that processed hot pot ingredients are cheap, but fresh vegetables and meat slices are relatively expensive, so he does not know how to choose. 【Learning content】 Regarding processed foods that are not true to their names, teach them how to avoid processed foods that are high in fat and salt and choose healthy and natural ingredients.
5. Drink enough water	【Understand】 1-7-1 Understand that drinking enough boiled water is important to health 1-7-2 Understand the influence of drinking too many sugar-sweetened beverages on health 【Analyse】 2-7-1 Analyse calories in commercial sugar-sweetened beverages 【Appraise】 3-7-1 Appraise whether an individual’s daily boiled water intake is sufficient	【Scenario】 The background of the story is the notion that college students drink sweetened beverages daily. Example: it was hot, and Ray felt thirsty and wanted a cold drink. Coincidentally, sweetened beverages are on sale. Ray bought a lot of beverages and planned to drink them slowly later. 【Learning content】 Focus on the problems that sweetened beverage cause for the human body and teach how to use ingenuity to make healthy drinks so that the body can absorb enough water.

Notes: NL, nutrition literacy; For nutrition literacy abilities (e.g. 1-1-1), the first number refers to the type: 1 = understand, 2 = analyse, 3 = appraise, and 4 = apply; the middle number refers to different health themes according to the Dietary Guidelines for Taiwanese; the third number corresponds to the serial number.

**Table 2:** Sample from the NL online intervention programme

Scenario-based video	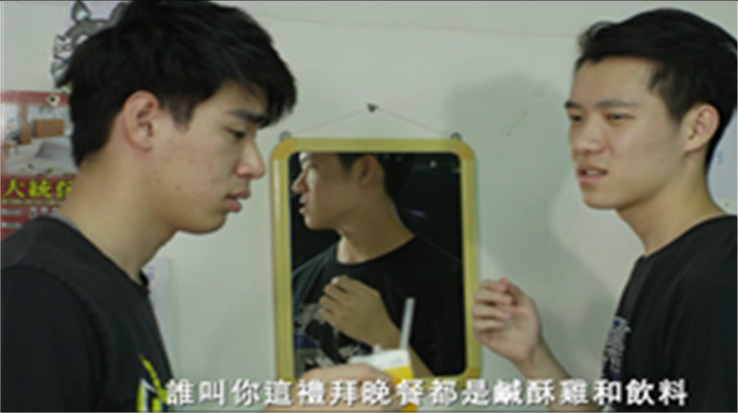	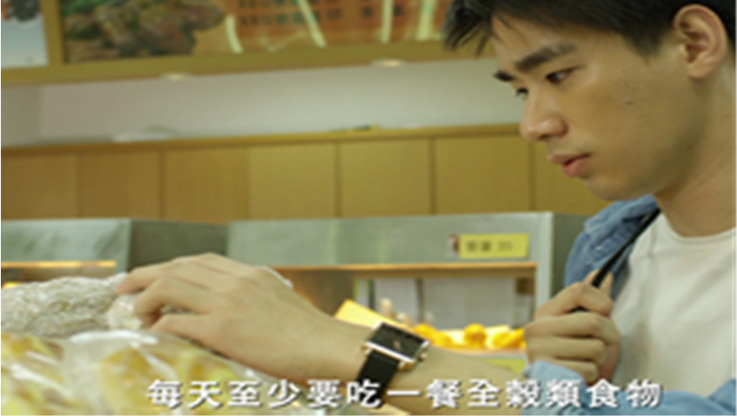
Subtitle	It’s your fault to have deep-fried chickens and sugar-sweetened beverages for dinner this week.	Eat at least one meal a day with whole grains
Learning content	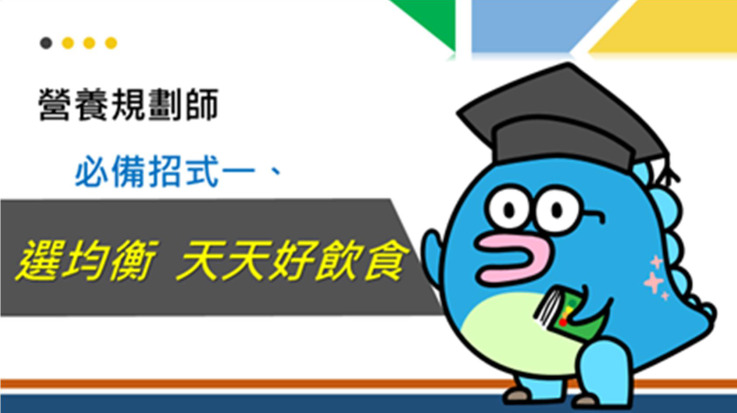	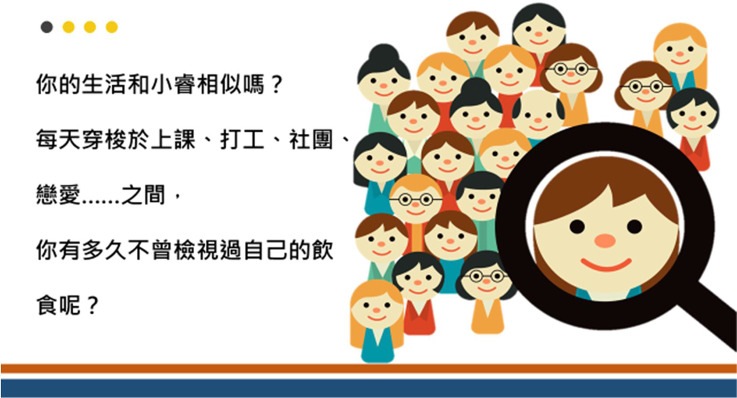
Content	First, choose a balanced diet every day	Is your life similar to that of the main character in the video?
Post-unit quiz	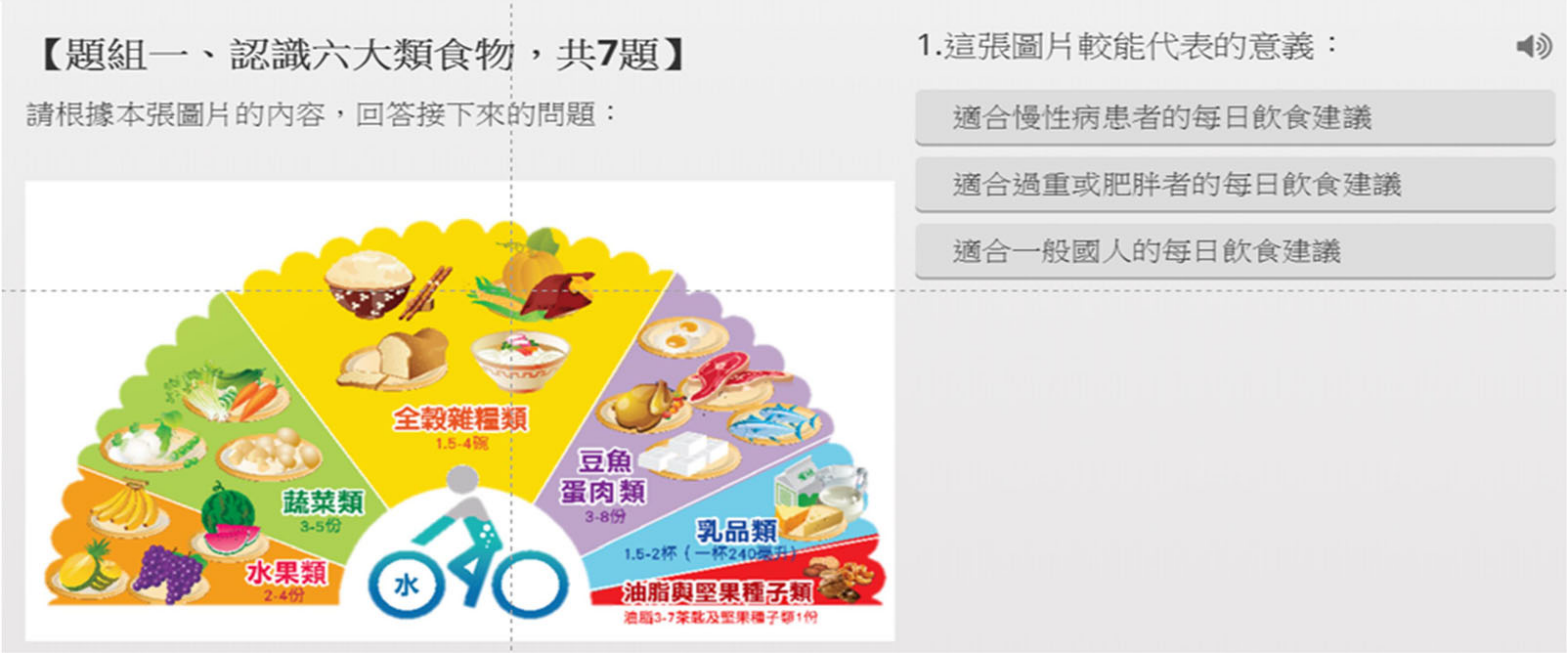	
Question	Q1. What is the meaning of this picture? A. Daily dietary recommendations suitable for the general population B. Daily dietary recommendations suitable for chronic disease patients C. Daily dietary recommendations suitable for overweight or obese people	

Notes: NL, nutrition literacy.

### Development process of the intervention

The NL online intervention programme was developed collaboratively by a diverse team, including health and nutrition education professors, middle school health education teachers, school dietitians, film crews from the university’s film and television department and network engineers from professional manufacturers. The programme’s framework and content were meticulously crafted, incorporating specific learning goals, scenario scripts and learning materials for each unit. These components were designed to align with the corresponding NL indicators, ensuring its effectiveness.

To ensure the programme’s relevance and applicability, a formative evaluation study was conducted. Feedback from a focus group of ten college students was obtained using McGuire’s information processing model^(37)^, which considers persuasive communication elements such as source, message, channel, receiver and destination. For example, questions like ‘What do you think of the professionalism and credibility of the intervention?’; ‘Do you like the overall design of the intervention?’; and ‘Do you think that using online platforms to deliver nutrition education aligns with your needs?’ were asked. Additionally, professionals in health and nutrition education reviewed the intervention plan, providing valuable insights on goal clarity, alignment with college students’ experiences and the overall design. Based on insights from formative research, the researcher produced real-life scenario-based videos. These were enhanced for authenticity by involving university students in enacting the scenarios and filming primarily on the campus.

To facilitate the learning process and provide easy access to materials, an online learning platform was developed and tested using Experience Application Programming Interface. This versatile e-learning specification allows for the collection of data in the form of activity statements, tracking participants’ progress and performance throughout various training and learning activities. The platform operates on both smartphones and computers, ensuring convenience for the learners.

### Evaluation of the intervention

#### Evaluation instrument

Two evaluation instruments were developed: an outcome evaluation instrument and a process evaluation instrument. The purpose of executing the outcome evaluation instrument was to evaluate whether NL and healthy eating behaviour had improved owing to the intervention of the online NL programme. The scales were developed as follows.

1. NL was measured with the Nutrition Literacy Measure for Taiwanese College Students^(34)^. The Nutrition Literacy Measure for Taiwanese College Students included two parts: a self-rated scale (NL scale) and a scenario-based test (NL test). The self-rated scale was developed based on the connotations of the eight principal indicators and included two items for *obtain* (e.g. For me, when there are nutrition-related issues, knowing where to find the right information is …), two items for *understand* (e.g. For me, to be able to understand the contents of Dietary Guidelines for Taiwanese is …), one item for *analyse* (For me, choosing foods from the nutritional point of view to distinguish food groups and functions is …), two items for *appraise* (e.g. For me, to judge whether the nutrition information on the network is correct or not is …) and one item for *apply* (For me, to use the right nutrition information in daily life for healthy eating is …). A four-point Likert scale ranging from 1 (*very difficult*) to 4 (*very easy*) was employed. The sum of all item scores is the self-rated NL scale score; higher scores reflect higher degrees of NL. The scenario-based test was formulated under the connotations of the 28 sub-indicators based on common dietary issues experienced by college students. It included thirty-two items across the eight scenarios. Multiple choice questions had three response options; only one option was correct. Each correct answer carried one point; the sum of item scores comprised the scenario-based NL test score. According to the results of the test in Taiwanese college students (*n* 1264)^(34)^, Cronbach’s *α* for the self-rated scale and the scenario-based test was 0·85 and 0·81, respectively. The measure showed a good model fit in two parts, and the difficulty parameters of the scenario-based test ranged from -3·22 to 0·11 in the item response theory analysis (acceptable range = -4 to 2). Furthermore, scores for two parts of the measure were both positively correlated with the healthy eating behaviour scale (*r* = 0·42, *P* <0·001 and *r* = 0·13, *P* < 0·001, respectively).
2. Healthy eating behaviour was measured using thirteen items on behaviours related to a *balanced diet* (eight items; e.g. I eat foods from each of the six food groups every day), *processed food consumption* (one item: When choosing foods, I try to select foods without artificial additives), *food label use* (three items; e.g. I read nutrition facts before I eat or buy foods) and *healthy food choice behaviour* (one item: I choose or modify my diet according to the principles of health). Participants were asked to rate how frequently they had engaged in these behaviours over the past week using a five-point Likert scale: 1 = *never* (0 d/week), 2 = *seldom* (1–2 d/week), 3 = *sometimes* (3–4 d/week), 4 = *often* (5–6 d/week), and 5 = *always* (7 d/week). The sum of all items represented the healthy eating behaviour score; higher scores indicated more frequent healthy eating behaviours. According to the results of a prior test with Taiwanese college students (*n* 1264)^(34)^, Cronbach’s *α* = 0·87, mean content validity index (CVI) = 0·99 and 2-week test-retest reliability intraclass correlation coefficient (ICC) = 0·49 (*P* < 0·001) for the entire scale.
3. Background data, including grade, age, sex, BMI category, parental education, residence, experience in taking nutrition-related courses and self-perceived need for access to nutrition information, were collected.

Process evaluation was performed only for the experimental group. The instrument was used to evaluate students’ participation, satisfaction and how helpful they found the online course to improve their NL after each unit. This evaluation used three items on a five-point scale ranging from 1 (*studying online courses not seriously at all*, *do not like it at all* or *not helpful at all*) to 5 (*studying online courses very seriously, like it very much* or *very helpful*). In addition, after completion of the five-unit online course, participants in the experimental group were asked to comment on the entire course in the post-test questionnaire using an open-ended question.

#### Data analysis

Independent sample *t*-tests and chi-square tests were used to assess differences in background variables between the groups. Paired sample *t*-tests were used to examine the within-group intervention effects between pretests and post-tests. Cohen’s *d* is a measure of effect size used to quantify the difference between two group means in SD units. It is calculated by dividing the difference between the means by the pooled SD of the two groups. According to Cohen’s conventions^(38)^, a value of 0·20 is considered small, 0·50 is medium, and 0·80 is large. We used ANCOVA, statistically controlling for the pretest results, to calculate and compare group differences in intervention effects. Eta squared (*η* ^2^) was used to measure the effect size between the groups. Eta squared is equivalent to *r* ^2^ and represents the proportion of total variance explained by an effect. According to Cohen’s conventions, eta squared value of < 0·01 is considered weak; 0·01 to < 0·059, small; 0·059 to < 0·138, medium; and ≥ 0·138, large. All statistical analyses were performed using SPSS (version 18.0; IBM Corp.).

## Results

### Participant characteristics

Among the participants, 81·6 % were female, and the average age was 19·88 years. Freshmen comprised the largest group (48·0 %) of participants, and the majority had a moderate BMI, with 23·5 % being overweight or obese and 14·3 % underweight. Regarding their parents’ highest level of education, the majority had completed high school or lower (63·8 %). Most of the participants resided in school dormitories (82·7 %), while only twenty-four students (24·5 %) indicated that they had taken a nutrition-related course in college. A total of seventy-six participants (77·6 %) believed that they needed nutrition knowledge or information. There were no significant differences between the experimental and control groups in any baseline characteristics (*P* value range: 0·159–1·000; Table 3).

**Table 3:** Demographic, BMI and prior knowledge characteristics of participants

Item	Experimental group *n* 49		Control group *n* 49		Total		*X* ^*2*^ */t*	*P*
	*n*	%	*n*	%	*n*	%		
Sex							0·00	1·000
Male	9	18·4	9	18·4	18	18·4		
Female	40	81·6	40	81·6	80	81·6		
	Mean	sd	Mean	sd	Mean	sd		
Age	19·94	1·4	19·82	1·2	19·88	1·3	0·46	0·650
	*n*	%	*n*	%	*n*	%		
Grade							0·88	0·829
Freshman	22	44·9	25	51·0	47	48·0		
Sophomore	10	20·4	10	20·4	20	20·4		
Junior	9	18·4	9	18·4	18	18·4		
Senior	8	16·3	5	10·2	13	13·3		
BMI							3·68	0·159
Underweight	10	20·4	4	8·2	14	14·3		
Normal	30	61·2	31	63·3	61	62·2		
Overweight or Obese	9	18·4	14	28·6	23	23·5		
Parental education							1·66	0·198
High school and below	27	57·4	33	70·2	60	63·8		
University and above	20	42·6	14	29·8	34	36·2		
Residency							0·64	0·424
Lived in school dormitory	39	79·6	42	85·7	81	82·7		
Lived off-campus or with family	10	20·4	7	14·3	17	17·3		
Had taken nutrition-related course							0·54	0·464
Yes	11	22·4	13	26·5	24	24·5		
No	36	73·5	30	61·2	66	67·3		
Need nutrition knowledge							0·09	0·955
Not much needed or not needed at all	11	22·4	11	22·4	22	22·4		
There is a need	32	65·3	31	63·3	63	64·3		
Needed very much	6	12·2	7	14·3	13	13·3		

Note. *X* ^*2*^ */t*: chi-square statistic/*t* value.

### Outcome evaluation

#### Effects within groups

Compared with the pre-intervention, participants in the experimental group showed significant post-intervention improvements in the NL test (*t* = -14·04, *P* < 0·001, *d* = 2·06), NL scale (*t* = -4·23, *P* < 0·001, *d* = 0·60) and healthy eating behaviour (*t* = -4·78, *P* < 0·001, *d* = 0·68) (Table 4). Those in the control group did not show any significant post-intervention improvements (control NL test, NL scale and healthy eating behaviour *P* values were 0·099, 0·244 and 0·126, respectively).

**Table 4:** Within-groups effects (paired *t* test)

Variable	Group	Pretest		Post-test		*t*	*P*	*d*
		*M*	*sd*	*M*	*sd*			
NL test	E	0·8	0·1	1·0	0·0	−14·04***	<0·001	2·06
	C	0·9	0·1	0·8	0·0	1·68	0·099	0·55
NL scale	E	4·3	1·0	4·9	0·7	−4·23***	<0·001	0·60
	C	4·3	1·1	4·5	0·9	−1·18	0·244	0·17
Healthy eating behaviour	E	3·2	0·7	3·5	0·5	−4·78***	<0·001	0·68
	C	3·0	0·7	3·2	0·7	−1·56	0·126	0·23

Note. NL, nutrition literacy; M, mean; *t*, *t* value; *d*, Cohen’s *d*; Group E, experimental group who received the online NL programme (*n* 49); Group C, control group who did not receive any nutrition education (*n* 49).

*** *P* value < 0·001.

#### Effects between groups

As the assumption of homogeneity of regression coefficients within groups was not violated (*P* > 0·05), ANCOVA was adopted to explore the effects between groups. The experimental group showed significant post-intervention improvements in the NL test (*F*_(1,95)_ = 86·06, *P* < 0·001, *η* ^2^ = 0·47), NL scale (*F*_(1,95)_ = 6·89, *P* = 0·01, *η* ^2^ = 0·12) and healthy eating behaviour (*F*_(1,95)_ = 4·84, *P* = 0·03, *η* ^2^ = 0·23) compared with the control group after controlling for pretest scores (Table 5).

**Table 5:** Between-group post-intervention effects (ANCOVA)

Variable	Source	SS	DF	MS	*F*	*P*	*η* ^2^
NL test	Group	0·7	1	0·7	86·06***	<0·001	0·47
	Error	0·7	95	0·0			
NL scale	Group	3·7	1	3·7	6·89*	0·010	0·12
	Error	51·4	95	0·5			
Healthy eating behaviour	Group	1·5	1	1·5	4·84*	0·030	0·23
	Error	30·3	95	0·3			

Note. NL, nutrition literacy; SS, sum of squares; DF, degrees of freedom, MS, mean square; *η* ^2^, effect size; Group E, experimental group who received an NL online programme (*n* 49); Group C, control group who did not receive any nutrition education (*n* 49).

* *P* value < 0·05.

*** *P* value < 0·001.

#### Process evaluation

In the experimental group, 89·8 % (*n* 44) of the participants indicated they engaged seriously/very seriously with the intervention curriculum, and 87·8 % (*n* 43) of them indicated that they liked the programme. Moreover, 98·0 % (*n* 48) of the participants found the intervention curriculum helpful or very helpful (Table 6). Students believed that the situational videos helped them connect to their personal life, the course design was lively and interesting, and the after-class quizzes allowed them to better understand their level of NL. They also looked forward to seeing the promotion of the online course to college students.

**Table 6:** Process evaluation of the NL online intervention programme (*n* 49)

	Very seriously		Seriously		Somewhat seriously		Not seriously		Not seriously at all	
	*n*	%	*n*	%	*n*	%	*n*	%	*n*	%
Overall, how seriously did you study this course?	17	34·7	27	55·1	4	8·2	1	2·0	0	0·0
	Liked it very much		Liked		Okay		Did not like		Did not like it at all	
Overall, how much did you like this course?	17	34·7	26	53·1	6	12·2	0	0·0	0	0·0
	Very helpful		Helpful		Okay		Not helpful		Not helpful at all	
Overall, do you think this course was helpful to you?	24	49·0	24	49·0	1	2·0	0	0·0	0	0·0

Note. NL, nutrition literacy.

## Discussion

We achieved the purpose of this study by developing and evaluating a scenario-based online NL programme that experimental group participants found interesting and helpful to meet the learning objectives. The results support the effectiveness of scenario-based online programmes for improving college students’ NL and eating behaviours.

NL does not merely refer to knowledge, but it also encompasses the ability to access and use nutritional information for making healthy food choices. Therefore, an NL-based nutrition education should be closely connected to students’ daily lives. When designing such interventions, it is essential to consider how the learning content fits into their everyday contexts. To do this effectively, a thorough understanding of the target audience’s needs is crucial before developing any intervention. Cousineau *et al.*ʼs study^(39)^ on web-based nutrition education for college students serves as an example of this approach. They utilised focus groups and concept mapping methodology to identify factors influencing food choices, difficulties in making healthy decisions and relevant suggestions for behaviour changes. In the present study, similar data collection methods involving focus groups were employed to ensure the programme’s relevance and immediate impact. However, it is important to acknowledge that further research and empirical evidence are required to establish a concrete and validated link between NL-based nutrition education and long-term improvements in dietary ability and personal dietary quality.

In Taiwan, despite formal health education courses being offered from elementary to high school, only 24·5 % of university students enrolled in nutrition-related courses, while 77·6 % expressed the need for nutrition knowledge in the present study in 2018. Higher education lacks formal health courses and relies on information campaigns and informal activities to improve health literacy. However, the impact of these interventions on healthy behaviours is limited^(40,41)^. With technological advancements and the COVID-19 pandemic, online education has become a mainstream alternative for health education, offering Internet-based nutrition education through websites, apps and online courses^(27)^. Previous studies have also indicated that website-based nutrition education has shown better effects compared to print and game modalities^(10)^ and the use of new technology can stimulate students’ interest in learning^(10,17)^. Similar to other nutrition interventions in college students^(27)^, online nutrition education has emerged as a viable means of providing nutrition education. Mounting evidence supports this approach, and our study not only confirms its effectiveness but also demonstrates the positive evaluation of the intervention programme during the process evaluation. These findings underscore the significance of extending the reach of such programmes, with technology-driven solutions proving more appropriate than other alternatives.

To enhance self-efficacy, some studies incorporate real-life situations as a teaching strategy in nutrition education programmes. Researchers have utilised methods such as using real food in experimental settings^(42)^, selecting food from photos or menus^(43)^ and employing buffets with food models^(44)^. However, these methods have their limitations, such as the high cost of materials, limited food choices and the gap between the experimental settings and the real-life food selection. Consequently, replications of these studies in real situations must be done with caution^(45,46)^. The integration of new technologies in scenario-based nutrition education could address these limitations. These technological advancements can bridge the gap between experimental settings and real-life situations, offering more practical and cost-effective solutions for nutrition education interventions. Furthermore, scenario-based learning in education holds promise for boosting self-efficacy and fostering successful experiences among learners^(21,24)^. Our study effectively integrated familiar campus scenes and diet-related problems as scenario-based elements to promote nutritional health. By applying this strategy to nutrition education, we aim to enhance learners’ capacity to modify unhealthy eating behaviours and make daily healthful decisions, which are essential for NL.

Additionally, in the experimental group, 10 % of participants (five individuals) reported varying degrees of seriousness towards the course, with four individuals moderately engaging and one person showing disinterest. Notably, participants were incentivised to complete the entire course to receive incentive money, reducing the likelihood of withdrawal. However, future researchers should focus on enhancing learners’ intrinsic motivation and concentration in the course. E-learning offers unlimited accessibility, openness and repeatability, which have advantages but may also diminish learners’ perception of the course value^(47)^. To address this, future studies could explore limiting accessibility and repeatability to create a sense of scarcity or exclusivity, potentially fostering increased engagement and concentration^(48)^. Considering such factors could lead to more effective and meaningful participation in online learning environments.

### Limitations and implications for research and practice

Our study had limitations. Firstly, using a convenience sample from a single university may restrict the generalisability of the findings. To improve validity and applicability, replicating the intervention at other educational institutions is crucial. Moreover, selecting two schools with comparable characteristics as experimental and control groups would enhance result validity by avoiding contamination. This approach ensures that the results are not influenced by factors unique to a particular university. Conducting the intervention in multiple schools would provide more comprehensive insights into its effectiveness.

Secondly, an unbalanced distribution of gender and grades could result in the underrepresentation of certain groups. Like other nutrition education intervention studies^(8)^, the current study had more female participants than male participants. For a more representative sample, future research should aim for a balanced representation of genders and academic levels.

Thirdly, this pilot study is the initial step in assessing the potential of a future full-scale project. To determine the intervention’s effectiveness, it is crucial to conduct larger studies with diverse student characteristics, a longer intervention duration, and include a delay effect evaluation, allowing for a comprehensive evaluation. Furthermore, to better assess the unique impact of the intervention and consider the effects of different instructional methods and delivery formats, it would be beneficial to include additional comparison groups. Incorporating a traditional nutrition education group and a face-to-face intervention group could provide valuable insights and contribute to a more comprehensive evaluation of the intervention’s effects.

Additionally, owing to the online course’s voluntary nature, it is likely that students who enrolled already possessed some interest in the topic. Consequently, the control group, despite not receiving any intervention, showed a slight improvement in their healthy eating behaviour from the pretest to post-test periods. Although this improvement lacked statistical significance, it suggests that the recruitment process and pretest may have influenced participants’ awareness of healthy eating. Careful interpretation of these findings is warranted. To strengthen evidence of the intervention’s effectiveness, researchers should secure adequate funding for a second wave of intervention targeting the same population. This follow-up study would yield more robust evidence regarding the intervention’s impact on the participants.

To enhance confidence in the evaluation results, we measured Nutrition Literacy Measure for Taiwanese College Students using a self-rated questionnaire and a scenario-based literacy test^(34)^. This combination of subjective and objective perspectives bolstered the intervention’s effectiveness. However, implementing NL confidently may remain challenging for some students. Future research can consider incorporating multiple perspectives to improve evaluation accuracy. Although significant NL and behavioural changes were observed, the relatively smaller effect size in behavioural change compared to the NL test requires further investigation. Simultaneously, it is crucial to consider the limitations posed by the possibility that participants may have provided socially desirable answers, particularly regarding their healthy eating behaviours. Conducting longer-term follow-up evaluations with more precise and objective dietary assessment tools will be essential to determine the intervention’s effectiveness in fostering consistent, long-term healthy eating habits.

This study addressed the developmental process of a scenario-based NL online programme for college students, incorporating NL indicators and real-life scenarios. Preliminary evidence was provided to support the effectiveness of the intervention. However, further research is required to establish stronger evidence. The positive outcomes found here encourage stakeholders to consider adopting this online scenario-based approach for promoting nutrition education in higher education. Continuous self-directed online learning can relieve pressure on school health staff and better cater to college students’ learning needs.
